# Combined Endoscopic Endonasal Transclival and Contralateral Transmaxillary Approach to the Petrous Apex and the Petroclival Synchondrosis: Working “Around the Corner” of the Internal Carotid Artery—Quantitative Anatomical Study and Clinical Applications

**DOI:** 10.3390/jcm13092713

**Published:** 2024-05-05

**Authors:** Carmine Antonio Donofrio, Francesco Corrivetti, Lucia Riccio, Sergio Corvino, Iacopo Dallan, Antonio Fioravanti, Matteo de Notaris

**Affiliations:** 1 Department of Neurosurgery, ASST Cremona, 2610 Cremona, Italy; carmine.donofrio@hotmail.com (C.A.D.); dott.luciariccio@libero.it (L.R.); antonio.fioravanti@asst-cremona.it (A.F.); 2 Division of Biology and Genetics, Department of Molecular and Translational Medicine, Faculty of Medicine, University of Brescia, 25121 Brescia, Italy; 3 Laboratory of Neuroscience, EBRIS Foundation, European Biomedical Research Institute of Salerno, 84125 Salerno, Italy; sercorvino@gmail.com (S.C.); matteodenotaris@gmail.com (M.d.N.); 4 Department of Neurosciences, Reproductive and Odontostomatological Sciences, Neurosurgical Clinic, School of Medicine, University of Naples “Federico II”, 80131 Naples, Italy; 5 Otorhinolaryngology, Audiology and Phoniatrics Operative Unit, Department of Surgical, Medical, Molecular Pathology and Emergency Medicine, Azienda Ospedaliero Universitaria Pisana, University of Pisa, 56124 Pisa, Italy; 6 Department of Neurosurgery, San Luca Hospital, Vallo della Lucania, 84078 Salerno, Italy; 7 Unit of Neurosurgery, University Hospital San Giovanni di Dio e Ruggi d’Aragona, University of Salerno, 84131 Salerno, Italy

**Keywords:** Caldwell–Luc, transmaxillary, multiportal approach, endoscopic, skull base, carotid artery, petroclival fissure

## Abstract

The endoscopic contralateral transmaxillary (CTM) approach has been proposed as a potential route to widen the corridor posterolateral to the internal carotid artery (ICA). In this study, we first refined the surgical technique of a combined multiportal endoscopic endonasal transclival (EETC) and CTM approach to the petrous apex (PA) and petroclival synchondrosis (PCS) in the dissection laboratory, and then validated its applications in a preliminary surgical series. The combined EETC and CTM approach was performed on three cadaver specimens based on four surgical steps: (1) the nasal, (2) the clival, (3) the maxillary and (4) the petrosal phases. The CTM provided a “*head-on trajectory*” to the PA and PCS and a *short distance to the surgical field* considerably furthering surgical maneuverability. The best operative set-up was achieved by introducing angled optics via the endonasal route and operative instruments via the transmaxillary corridor exploiting the advantages of a non-coaxial multiportal surgery. Clinical applications of the combined EETC and CTM approach were reported in three cases, a clival chordoma and two giant pituitary adenomas. The present translational study explores the safety and feasibility of a combined multiportal EETC and CTM approach to access the petroclival region though different corridors.

## 1. Introduction

The petrous apex (PA) and petroclival synchondrosis (PCS) have always represented challenging surgical targets, regardless of the preferred approach: transcranial or endoscopic endonasal (EEA). The control of the internal carotid artery (ICA) is a milestone for coronal modules of EEAs [1,2,3]. EEAs to access the PA and PCS are laterally limited by the petrous (ptICA) and paraclival (pcICA) segments of the ICA. The *medial trans-pterygoid* approach with ICA lateralization and the *trans-pterygoid infrapetrous* approach, selected according to tumour extension, biology and relationships with the ICA, are the main expanded EEAs (EEEA) proposed to go one step beyond this anatomical boundary, expanding the ventral route for deep paramedian skull base pathologies [1,3,4,5,6,7,8]. However, a long-lasting learning curve is required to handle these technically demanding EEEAs that present significant postoperative morbidity rates, even in expert hands.

Combined endoscopic skull base approaches–endonasal, transmaxillary, and transorbital–gained popularity in recent years because of better knowledge of endoscopic anatomy, which proved the advantages of multiportal surgery, particularly in terms of visualization and surgical freedom, and reduction of postoperative morbidities [7,8,9,10,11,12,13]. The endoscopic contralateral transmaxillary (CTM) approach has been described as a potential route to widen the corridor posterolateral to the ICA [3,7,8,10,12].

This paper is the result of a reverse translational study [14]. We refined the surgical technique of a combined multiportal endoscopic endonasal transclival (EETC) and CTM approach to the PA and PCS in the dissection laboratory, exploring its advantages and limitations before moving to the operating room. We then reported our preliminary surgical experience.

## 2. Materials and Methods

### 2.1. Anatomical Study

Surgical dissections were realized on both sides of three cadaver specimens embalmed and injected with coloured latex solutions atLaboratory of Neuroanatomy of the EBRIS Foundation in Salerno.. Microsurgical dissections were performed using dedicated skull base surgical instruments under endoscopic visualization (HOPKINS II, 4 mm by 18 cm, 0° and 30° optics; Karl Storz, Culver City, CA, USA). Bone drilling was performed with a high-speed electric drill (Midas Rex; Medtronic Inc., Dublin, Ireland) equipped with cutting and diamond burrs.

### 2.2. Surgical Technique

The head was slightly extended, 15° rotated towards the surgeon and secured in a three-pin head holder. The combined EETC and CTM approach was based on four surgical steps: (1) *nasal*, (2) *clival*, (3) *maxillary* and (4) *petrosal* phases.

(1) The nasal step

**Figure 1:**
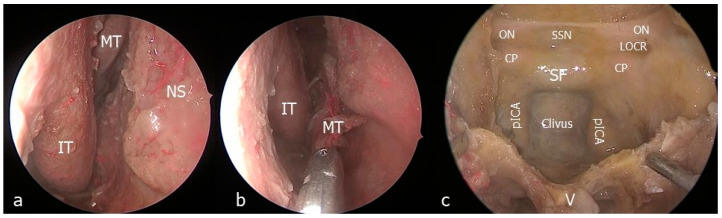
Endonasal step. (**a**) Endoscopic view of the right nostril; (**b**) Middle turbinectomy; (**c**) Sphenoid sinus exposure. CP: clinoidal process; IT: inferior turbinate; LOCR: lateral optico-carotid recess; MT: middle turbinate; ON: optic nerve; pcICA: paraclival segment of the internal carotid artery; SSN: suprasellar notch; V: vomer.

Middle and inferior turbinectomies were achieved bilaterally, keeping the head of the inferior turbinate to preserve the nasolacrimal duct. An endonasal medial maxillectomy extending from the posterior wall of the maxillary sinus to the nasolacrimal canal was then performed on the side opposite to the surgical target. A nasoseptal mucosal flap pedicled on the posterior septal artery was harvested and stored in the nasopharynx. Once completed, the posterior septectomy, bilateral posterior ethmoidectomy and expanded transrostral sphenoidotomy were accomplished (Figure 1).

(2) The clival step

**Figure 2:**
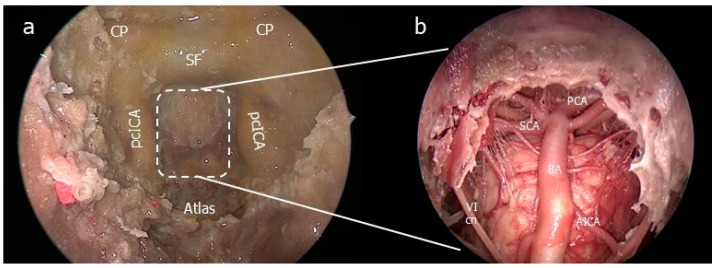
Endonasal step. (**a**) Clivectomy. (**b**) Endoscopic intradural visualization. AICA: anteroinferior cerebellar artery; CP: clinoidal process; PCA: posterior cerebral artery; pcICA: paraclival segment of the internal carotid artery; SCA: superior cerebellar artery; SF: sellar floor.

The sphenoidal septations were reduced, the sphenoidal mucosa were exenterated and the intrasphenoidal landmarks were identified depending on the sinus pneumatisation [13,15]. After dissecting nasopharyngeal mucosa and basopharyngeal fascia, the sphenoidal floor was grounded to the plane of the clival recess. The clivus was then drilled to the depth of the posterior cortical bone as wide as possible to the pcICA bilaterally. The medial aspect of the pcICA was exposed by removing the bone of the lateral wall of the clivus (Figure 2).

(3) The maxillary step

**Figure 3:**
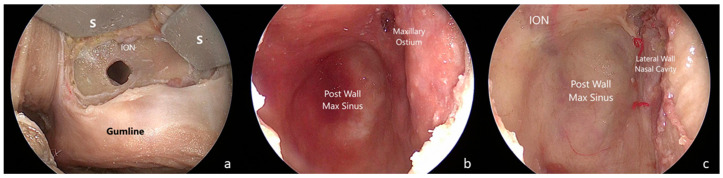
Sublabial-transmaxillary approach. (**a**) Sublabial exposure of the canine fossa, acceding to the maxillary sinus drilling the anterior wall below the inferior orbital nerve. (**b**) Endoscopic view of the maxillary sinus. (**c**) Endoscopic view of the maxillary sinus after removal of the mucosa. ION: inferior orbital nerve; S: spatula.

The maxillary step was completed realizing a Caldwell–Luc approach. An anterior 2 × 2 cm maxillectomy extended laterally to the zygomatic maxillary process and superiorly to the infraorbital foramen was performed through a linear incision at the bucco-gingival sulcus [7,16,17]. The maxillary mucosa was dissected and the medial maxillectomy flattened to the level of the nasal floor [12] (Figure 3).

(4) The petrosal step

**Figure 4:**
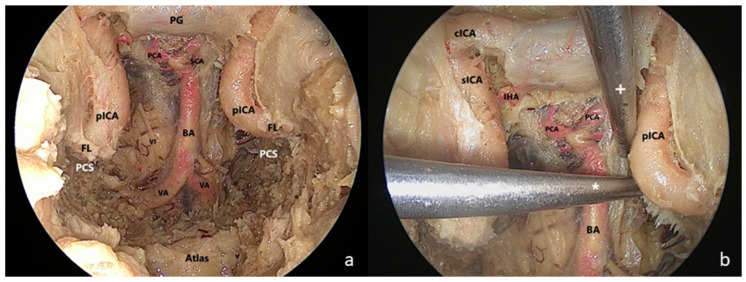
Endoscopic endonsal transsphenoidal approach with extended exposure of the ventral skull base. (**a**) The clivus was completely drilled out and the paraclival segment of the internal carotid artery (pcICA) was exposed bilaterally. The medial petrous apex was drilled up to the foramen lacerum (FL) inferiorly, providing access to the petroclival synchondrosis (PCS). The clival dura was then removed to identify intradural landmarks. (**b**) Two straight surgical aspirators were inserted through the endonasal (+) and transmaxillary (*) corridor showing the different angles of attack toward the PCS; BA: basilar artery; FL: foramen lacerum; PCA: posterior cerebral artery; PCS: petroclival synchondrosis; PG: pituitary gland; pICA: paraclival segment of the internal carotid artery; SCA: superior cerebellar artery; VA: vertebral artery; VI: sixth cranial nerve.

A “two-surgeon four–hand technique” was adopted. The medial PA was drilled up to the clival dura medially, the foramen lacerum (FL) inferiorly, the pcICA antero-laterally, the petrous bone postero-laterally and the cavernous sinus superiorly. The ‘carotid-clival window’ was opened, providing access to the PCS [18]. The clival dura was incised along the midline in a T shape for identifying intradural landmarks (Figure 4).

### 2.3. Quantitative Analysis and Surgical Operability

PA and PCS were approached through the contralateral endonasal and transmaxillary corridors using both straight and angled optics and instruments. The *angles of attack* between the trajectory of the surgical corridors and the major axis of the PA, the *surgical field depths* between the operative windows–nostrils and anterior maxillectomy–and the PA, and the *petrous drilling depths* between the PA and the posterior limits of the petrous bone drilling were measured on CT through neuronavigation (StealthStation S8; Medtronic Inc, Dublin, Ireland) for both the endonasal and transmaxillary routes [7,8,12,17]. Surgical operability was assessed as *surgical exposure* and *maneuverability* on a scale from 0 to 2, as previously described [17,19,20].

### 2.4. Statistical Analysis

Quantitative variables were expressed as means (±standard deviation) and compared with the unpaired Student’s *t*-test. A threshold of two-tailed probability (*p*) value ≤ 0.05 was set for statistical significance. All the analyses were performed with SPSS 23.0 (SPSS Inc., Chicago, IL, USA).

### 2.5. Surgical Series

Clinical cases are described to translate our findings into a real surgical scenario. Three patients presenting with complex petroclival lesions extended towards the PA and PCS underwent a combined EETC and CTM approach. Standard written informed consent was obtained from all patients. Management of patients’ data was conducted in accordance with the ethical standards of the institutional and national research committee and with the 1964 Declaration of Helsinki and its later amendments.

## 3. Results

### 3.1. Anthropometric Measurements and Surgical Operability

**Figure 5:**
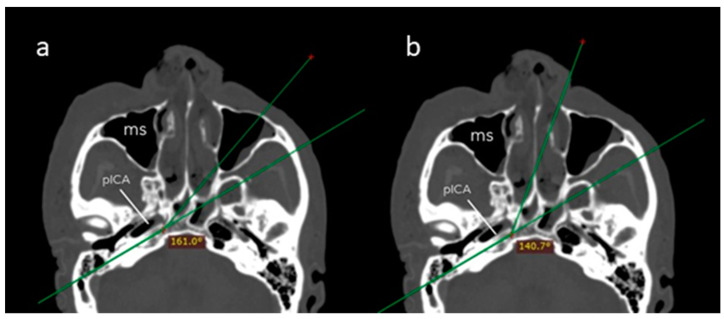
Axial CT scan showing the angle of attack of the endoscopic endonasal (**a**) and contralateral transmaxillary (**b**) approaches. A line parallel to the axis of the petrous segment of the internal carotid artery is used as a reference to calculate the angles of attack. MS: maxillary sinus.

The *angle of attack* was significantly wider across the transmaxillary than the endonasal route (161.1° ± 8.6° vs. 140.6° ± 5.7°; *p* = 0.001). The mean gain of angle of attack through the transmaxillary corridor was 20.5° (±4.7°), affording a surgical trajectory more parallel to the major axis of the PA (Figure 5).

As detailed in Table 1, *surgical filed depths* were significantly shorter (74.3 ± 4.0 vs. 88.0 ± 5.8 mm; *p* = 0.001) and *petrous drilling depths* were significantly deeper (17.2 ± 4.0 vs. 11.0 ± 3.3 mm; *p* = 0.008) through the transmaxillary corridor.

*Surgical exposure* was limited (1) using straight optics as *surgical maneuverability* was constrained (2), passing both straight and angled instruments through the endonasal corridor. The best set-up for two-surgeon–four-hand surgical dissections was achieved by introducing angled optics through the contralateral endonasal route and operative instruments through the transmaxillary corridor (Table 2).

### 3.2. Surgical Series

Case 1

**Figure 6:**
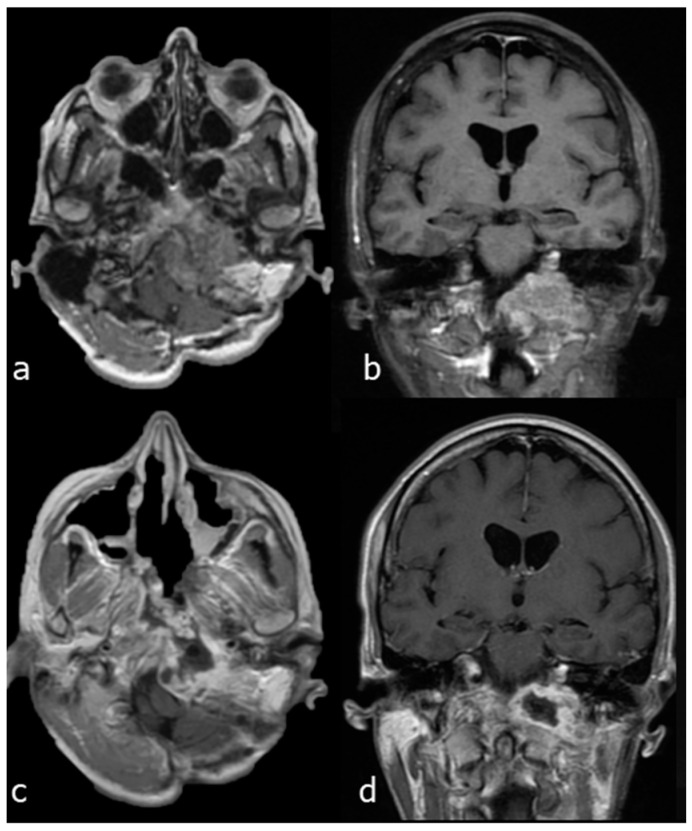
Case 1: preoperative and postoperative MRI scans of a large cranio-cervical junction chordoma, underwent first surgery through a retrosigmoid craniotomy, and then operated on by a combined EETC and CTM approach. (**a**) preoperative MRI scan, axial view. (**b**) preoperative MRI scan, coronal view. (**c**) postoperative MRI scan, axial view. (**d**) postoperative MRI scan, coronal view.

A 44-year-old woman affected by a large cranio-cervical junction chordoma subtotally resected through a retrosigmoid craniotomy 5 years before presented a slowly progressive tumour recurrence. A contrast-enhanced brain MRI showed a significant bilateral invasion of the petroclival region, with remarkable brainstem compression and displacement, and an extension towards the left parapharyngeal space. The patient presented with dysphonia and mild dysphagia. The tumour was approached through a combined EETC, left trans-pterygoid and CTM approach. The pcICA was exposed on the left side. Tumour resection was started through the endonasal corridor to remove the brainstem extradural component of the lesion. Then, the left parapharyngeal tumour portion was accessed using the endonasal corridor for endoscopic visualization and the transmaxillary route for surgical resection. A right nasoseptal flap was used for skull base reconstruction. Near total resection was obtained, as shown by the postoperative MRI (Figure 6).

Case 2

**Figure 7:**
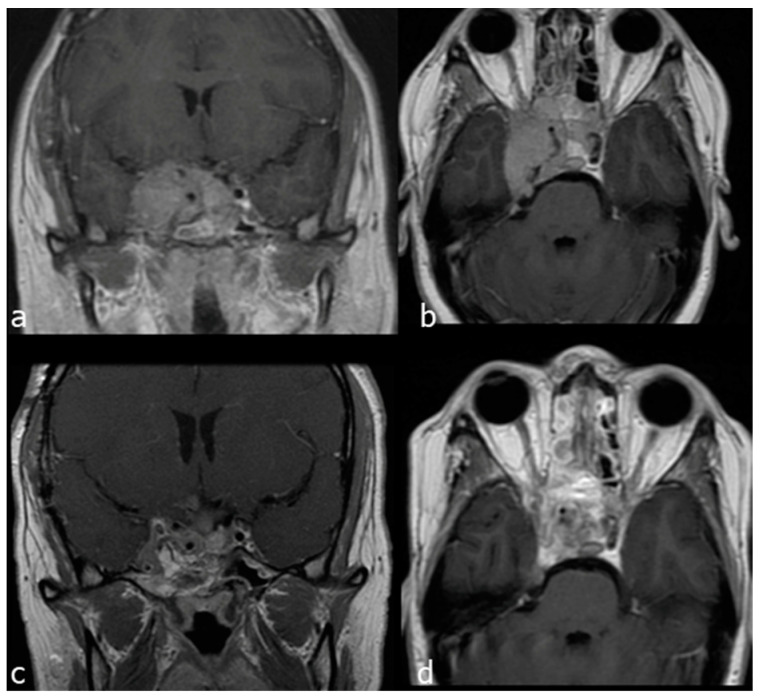
Case 2: preoperative and postoperative MRI scans of a recurrent giant non-secreting pituitary adenoma, previously operated through transcranial and then operated on by a combined EETC and CTM approach. (**a**) preoperative MRI scan, axial view. (**b**) preoperative MRI scan coronal view. (**c**) postoperative MRI scan, axial view. (**d**) postoperative MRI scan, coronal view.

A 28-year-old woman affected by a recurrent giant non-secreting pituitary adenoma, previously operated through transcranial and EEA approaches, presented an extensive invasion of the right cavernous sinus (Knosp grade IV), causing trigeminal compression and facial numbness. She underwent a combined EETC, right trans-pterygoid and CTM approach. An indocyanine green video-angiography was performed intraoperatively to locate the pcICA [21]. The sellar portion of the tumor was removed through the endonasal corridor, and the CTM was exploited to resect the tumour invading the right cavernous sinus, laterally to the cavernous segment of the ICA. A multilayer reconstruction with fat, fascia lata and a right nasoseptal flap was then performed. Near total resection was accomplished (Figure 7).

Case 3

**Figure 8:**
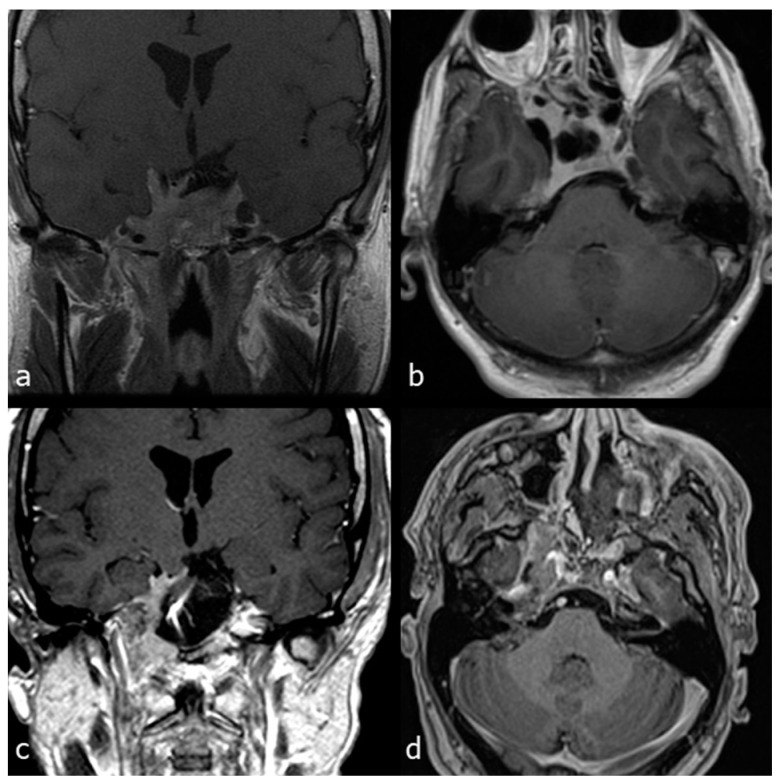
Case 3: preoperative and postoperative MRI scans of a recurrent giant non-secreting pituitary adenoma, previously operated through transcranial and then operated on by a combined EETC and CTM approach. (**a**) preoperative MRI scan coronal view. (**b**) preoperative MRI scan, sagittal view. (**c**) postoperative MRI scan, coronal view. (**d**) postoperative MRI scan, sagittal view.

A 39-year-old man affected by a recurrent giant prolactin-secreting pituitary adenoma with clival extension, bilateral cavernous sinus invasion mainly on the right side and displacement of the cavernous segment of the ICA was referred to surgery because of dopamine agonists’ resistance causing tumour growth and visual worsening. He underwent a combined EETC and CTM approach and a right nasoseptal flap was raised for reconstruction. Subtotal resection was achieved by removing the clival and right cavernous sinus tumor components (Figure 8).

## 4. Discussion

We reviewed the relevant surgical anatomy, advantages, drawbacks and indications of the combined EETC and CTM approach to the PA and PCS.

### 4.1. Surgical Anatomy Considerations

A thorough understanding of the endoscopic anatomy of the ICA and its landmarks is crucial to performing this multiportal approach and evaluating its advantages compared to the EEEAs.

Six segments of the ICA have been described from the endoscopic perspective: *parapharyngeal*, *petrous*, *paraclival*, *parasellar*, *paraclinoid* and *intradural* [2,15]. The ptICA runs in an inferior-to-superior, posterior-to-anterior and lateral-to-medial trajectory. The ptICA comprises three parts: the vertical portion, the posterior genu and the horizontal portion. After exiting the carotid canal, the horizontal portion of the ptICA continues as the pcICA forming the anterior genu, sited at the FL, before climbing upwards, where it lies medially to the inferior aspect of the Gasserian ganglion within the Meckel’s cave [2,5,15].

The ptICA and pcICA are in close spatial relationships with several anatomical structures that can be grouped in: (1) extracranial—the *vidian canal* and the *Eustachian tube*; (2) bony—the *FL*, the *PCS* and the *PA*; and (3) intradural—the *deep petrosal* and *abducens nerves*.

The *vidian canal* is located at the junction of the medial pterygoid plate and the floor of the sphenoid sinus. It opens anteriorly into the medial part of the posterior wall of the pterygopalatine fossa and posteriorly at the level of the anterolateral edge of the FL, representing a crucial endoscopic landmark for the anterior genu of the pcICA [15,22,23]. The vidian canal is coursed by the vidian nerve that carries pre-ganglionic parasympathetic fibers—contribution of the greater petrosal nerve—and post-ganglionic sympathetic fibers—contribution of the deep petrosal nerve—to the pterygopalatine ganglion. The *Eustachian tube* lies anterolaterally and roughly parallel to the horizontal portion of the ptICA, following an inferior-to-superior and lateral-to-medial oblique course [2,8,23,24]. The vidian–eustachian junction is a reliable and specific landmark of the anterior genu of the pcICA at the level of FL [23]. The *FL* is formed by the confluence of the synchondroses between the sphenoid bone, the PA of the temporal bone and the clival portion of the occipital bone. The pterygosphenoidal fissure runs anteromedially, the petrosphenoidal fissure runs posterolaterally and the PCS runs posteromedially to the FL [2,15,25,26]. The *PCS* is located medially to the pcICA and turns inferolaterally toward the jugular foramen, accommodating the inferior petrosal sinus [26,27,28]. The *PA* of the temporal bone is pyramidal and is composed of the apex, which points anteromedially and confines anteromedially with the posteroinferior portion of the cavernous sinus and posterolaterally with the trigeminal nerve; the base, which is directed posterolaterally and is delimited by a virtual line passing through the anterior border of the internal acoustic meatus; the superior surface, part of the middle cranial fossa floor laterally bordered by the horizontal segment of the ptICA, the anterior genu of the pcICA and the FL, and the inferior surface, the anterior third of the posterior petrous bone surface that joins at the petrous ridge, where the superior petrosal sinus courses [8,27,28].

The *deep petrosal nerve* arises from the *internal carotid plexus*, enters the skull via the carotid canal and joins with the greater petrosal nerve to form the vidian nerve, carrying post-ganglionic sympathetic fibres originating from the superior cervical ganglion [29,30]. The *abducens nerve* lies at the upper PCS, where it turns from a vertical direction of the cisternal and gulfar segments to a more horizontal direction of the cavernous segment [15,26,31].

### 4.2. Advantages and Drawbacks of the Combined EETC and CTM Approach

The advantages and drawbacks of the combined EETC and CTM approach compared to the EEEAs to access the PA and the PCS can be analysed in terms of *surgical exposure*, *maneuverability* and potential *postoperative morbidities*.

**Figure 9:**
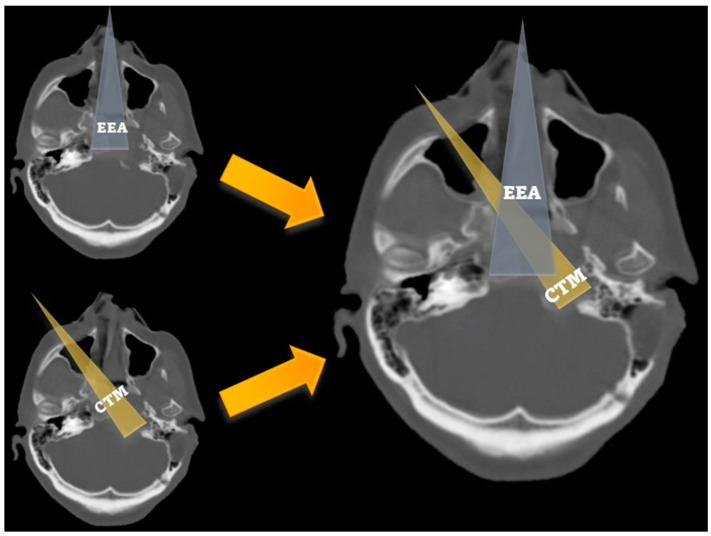
Bone CT scan comparing the trajectories and the area of exposure related to the endoscopic endonasal (grey) and the contralateral transmaxillary (yellow) approaches, showing that the endonansal corridor provides a good exposure of the medial clivus, while contralateral transmaxillary corridos allow reaching the lateral clivus and the petroclival synchondrosis.

As reported in the literature and confirmed by our findings, the combined EETC and CTM approach affords a significant increase in the angle of attack (between 20° and 25°) relative to the major axis of the PA, exploiting the transmaxillary corridor [7,8,9,10,12]. Moreover, we described significantly shorter surgical field depths across the transmaxillary compared to the endonasal corridor [8]. Overall, the CTM provides a “*head-on trajectory*” to the PA and PCS and a *short distance between the pivot point* of endoscopic instruments *and the surgical field,* considerably furthering surgical maneuverability [7,8,10,12,17]. Consequently, the petrous drilling can be deeper using the transmaxillary as working corridor (Figure 9).

We described a “two-surgeon–four-hand” technique placing angled optics via the endonasal route and operative instruments via the transmaxillary corridor, improving surgical freedom and microdissection dexterity [10]. This multiportal approach circumvents the constraints of positioning multiple instruments and optics through the endonasal corridors and the challenges of working with angled optics and instruments. Preventing the “sword-fighting” and “fisheye” effects enhances the surgical control of critical structures, particularly in case of intraoperative complications’ occurrence, such as ICA injuries [3,10,12,17,32,33].

The multiportal EETC and CTM approach avoids manipulating anatomical structures surrounding the surgical targets, reducing the risk of consequent morbidities. This approach specifically safeguards the integrity of pterygopalatine fossa, vidian nerve and Eustachian tube, and minimizes mobilization of the ICA, especially if compared to the trans-pterygoid EEEAs.

The pterygopalatine fossa is not violated preserving the sphenopalatine artery and vascular supply of the nasoseptal flap, which can be used for treating CSF leakage and protecting exposed neurovascular structures, especially in cases that require adjuvant radiotherapy [7,8,10,12]. The vidian nerve and the Eustachian tube are well-kept, avoiding the “dry eye and nose” syndromes, middle ear effusions, recurrent otitis media and hearing loss [5,8,34]. There is no need for the skeletonizing or mobilization of ptICA and pcICA segments, which are technically challenging and risky maneuvers that require constant and dynamic retraction of the vessel, potentially causing direct and indirect injuries of ICA, greater petrosal, deep petrosal and vidian nerves [2,6,7,8,10,12,26]. Moreover, anatomical variants, such as dehiscence of the overlaying bone or unexpected course of the ICA, previous surgeries, causing scar tissue formation, or radiation treatments, inducing vessel wall weakening, may increase the risk of intraoperative ICA injury when its extensive manipulation is required [3,7,15].

On the other hand, morbidities related to the CTM approach are usually transient and include facial swelling, asymmetry, numbness and/or paraesthesias; oro-antral fistulas; dental devitalization; and dacryocystitis due to nasolacrimal duct stenosis [8,10,12,16,17].

### 4.3. Surgical Considerations

Over the last twenty years, different transcranial, endonasal and transfacial routes have been described to access the lateral aspect of the petroclival region behind the ptICA, which represents the main anatomical barrier for all of them. More recently, transmaxillary approaches have been described to improve the ventral route of EEEA and reach “far away” paramedian anatomical regions (i.e., the petroclival area, jugular foramen), providing better angles of attack of modular combined approaches. These multiportal non-coaxial approaches allow the surgeon to exploit different endoscopic and working trajectories gaining the advantage of “seeing behind” and “working around” the anatomical structures that cross the surgical field.

In this scenario, the multiportal combined EETC and CTM approach has been used to access the PA and PCS far laterally and posteriorly to the ptICA and pcICA. This technique has been adapted especially to approach tumors of the PA and PCS such as chordomas, chondrosarcomas, cholesterol granulomas, metastases and meningiomas [7,8,10]. We reported our preliminary surgical experiences of combined EETC and CTM approach to treat petroclival chordomas and giant pituitary adenomas extending laterally to the ICA that have always represented a surgical challenge regardless of endonasal or transcranial approaches [35,36,37,38,39]. Our study demonstrates the safety and feasibility of this approach. The use of multiple operative corridors obviates the need for large operative spaces avoiding extensive manipulations and preserving the surrounding anatomical structures, thus minimizing the related postoperative complication and morbidity rates. The possibility of safely working behind the ICA may reduce the need for second surgeries and improve the extent of tumor resection. However, alternative transcranial or transfacial approaches should be considered according to anatomical variants and tumor extension, biology and relationships with neurovascular structures.

## 5. Conclusions

The present reverse translational study explores the safety and feasibility of a combined multiportal EETC and CTM approach to access the petroclival region through different corridors without crossing the cranial nerves’ plane and avoiding the ICA and brain manipulations. However, other surgical series are needed to confirm our findings and validate this technique as a valuable option to consider for modular multiportal paramedian skull base approaches.

## Figures and Tables

**Table 1:** Anthropometric measurements through the contralateral transmaxillary and endonasal corridors.

	Transmaxillary Corridor (Mean ± SD)	Endonasal Corridor (Mean ± SD)	*p* Value
Angle of attack (°)	161.1 (8.6)	140.6 (5.7)	0.001
Surgical field depth (mm)	74.3 (4.0)	88.0 (5.8)	0.001
Petrous drilling depth (mm)	17.2 (4.0)	11.0 (3.3)	0.008

**Table 2:** Surgical operability around the petrous apex and the petroclival fissure through the contralateral transmaxillary and endonasal corridors.

	Surgical Exposure		Surgical Maneuverability	
	0° Optics	30° Optics	Straight Instruments	Angled Instruments
Transmaxillary corridor	2	2	2	2
Endonasal corridor	1	2	1	1

## Data Availability

The data presented in this study are available on request from the corresponding author due to ethical reason.
